# The Removal of Pollutants from Wastewater Using Magnetic Biochar: A Scientometric and Visualization Analysis

**DOI:** 10.3390/molecules28155840

**Published:** 2023-08-03

**Authors:** Chenyang Li, Chongbin Zhang, Shuang Zhong, Jing Duan, Ming Li, Yan Shi

**Affiliations:** 1 Key Laboratory of Songliao Aquatic Environment Ministry of Education, School of Municipal and Environmental Engineering, Jilin Jianzhu University, Changchun 130118, China; lichenyang0331@126.com (C.L.); zhangchongbin1999@163.com (C.Z.); 2 Key Laboratory of Groundwater Resources and Environment, Ministry of Education, Jilin University, Changchun 130021, China; zhongshuang@jlu.edu.cn; 3 Huaneng Songyuan Thermal Power Plant, Songyuan 138000, China; yjj980619@163.com; 4 Jilin Academy of Agricultural Sciences, Changchun 130033, China

**Keywords:** visualization, contaminants, magnetic biochar, bibliometric analysis, mechanisms

## Abstract

In recent years, the use of magnetic biochar in wastewater treatment has shown significant effects and attracted scholars’ attention. However, due to the relatively short research time and the lack of systematic summaries, it is difficult to provide a more in-depth analysis. This study utilizes RStudio and CiteSpace software to comprehensively analyze the research trends and progress of magnetic biochar in wastewater treatment. The analysis of bibliometrics is performed on 551 relevant papers retrieved from the Web of Science, spanning the period between 2011 and 2022. The most influential countries, institutions, journals, disciplinary distribution, and top 10 authors and papers in this field have been identified. The latest dataset has been used for keyword clustering and burst analysis. The results indicated that: (1) Bin Gao is the most influential author in this field, and high-level journals such as Bioresource Technology are more inclined to publish articles in the field of magnetic biochar. (2) Research in this field has predominantly focused on the removal of heavy metals and organic compounds. Keyword burst analysis shows a shift in research direction towards the removal of complex organic pollutants recently. (3) For the future development of magnetic biochar, an environment-friendly approach, economic viability, and joint technology are the directions that need more exploration. Finally, this paper provides a summary of the various adsorption mechanisms of magnetic biochar and several common modification methods, aiming to assist scholars in their research endeavors.

## 1. Introduction

More than 70% of the world’s surface is covered by water, but only a tiny part of this is available for human use [1]. Unfortunately, a growing portion of these available water resources have become contaminated with heavy metals, polyaromatic hydrocarbons, dyes, and other pollutants [2]. The pollution in water environments poses a considerable threat to human health, with more than 2 billion people worldwide suffering from waterborne diseases [3,4]. Studies have shown harmful metals can accumulate in the body even at low levels, causing high blood pressure, anemia, nervous system disorders, and red blood cell damage [5]. Residual antibiotics pose a serious threat to ecosystems [6]. Most antibiotics are weakly absorbed in the body and are excreted into the environment. As reported, 25~90% of antibiotics are returning to the environment in the form of parent compounds or metabolites, which is significantly beyond the capacity of ecosystem biodegradation [7]. Therefore, various technologies such as physical, chemical, biological, and physical-chemical methods have been developed and implemented to remove pollutants in sewage [8,9]. Among these, adsorption technology has gained considerable attention due to its ability to efficiently separate substances in both liquid and solid phases [10]. Adsorption technology offers advantages such as easy processing, cost effectiveness, and high effectiveness [11], making it the most commonly used approach for sewage treatment.

Commonly used adsorbents include activated carbon [12], silica gel [13], zeolite [11], and clay minerals [14]. However, due to the high cost, using these materials on an industrial scale is difficult. Biochar (BC), on the other hand, is a carbon-rich material that offers a low-density, stable, and porous structure [15]. It is derived from biomass residues, such as agricultural and forestry waste materials, through pyrolysis [16,17]. Due to its porous structure, excellent physical and chemical properties, large specific surface area, rich mineral composition, and surface functional groups, biochar is considered an environmentally friendly adsorbent [18,19].

While biochar has shown promise in adsorbing heavy metals from water [20], the practical utilization of biochar is currently hindered by several challenges. Firstly, biochar faces issues with recovery and regeneration, leading to inefficient use [21]. Moreover, its small particle size and low density make it difficult to separate from water [22]. To address this, a promising approach is to combine biochar with magnetic materials, allowing for magnetic separation [23,24]. For instance, metal materials such as iron (Fe), magnesium (Mg), cobalt (Co), nickel (Ni), and zinc (Zn) can be loaded onto the surface of biochar [25], and magnetic biochar composites can be prepared using metal salts or metal oxides.

There are various methods for preparing magnetic biochar with three main typical methods [26]: impregnation, precipitation, and activation. Mubarak et al. [27] used FeCl_3_ solution impregnation and microwave heating to obtain Fe_3_O_4_-containing biochar from fruit branch powder. Sarswat and Mohan et al. [28,29] used precipitation methods to ground biochar raw material into powder, added Fe^2+^/Fe^3+^ solution, and then gradually added NaOH (25% *v*/*v*) solution to a pH value of 10–11. After filtration, washing, and drying, the magnetic biochar containing FeSO_4_ was obtained. Activation methods include physical, chemical, and combined physical–chemical activation methods. In this approach, the oxygen-containing function groups of the biochar surface is activated by active substances, the micropore structure is improved, and the porosity and specific surface area are increased [30], thereby improving the overall adsorption capacity and kinetic performance. The yield of magnetic biocarbon produced through activation varies significantly depending on the specific method employed. Compared to a simple carbonization process, the activated carbon content may be reduced [29], but the hydrophilicity is better and the specific surface area significantly increases. In the application of magnetic biochar, temperature, adsorbent dosage, pH value, time, and pollutant concentration can determine the application effect [31]. With good adsorption capacity for heavy metal ions in water, magnetic biochar can be separated from particles in water by external magnetic force [32,33]. Moreover, it has been widely used in the treatment of water pollutants and the separation of pollutants. Recent research found that adsorption was only a part of the performance of magnetic biochar. Catalysis has also played a dominant role in the field of magnetic biochar. Magnetic biochar can not only remove pollutants in water by adsorption but also use catalysis to degrade pollutants in water to achieve water purification. For example, Rong et al. [34] synthesized a new type of magnetic biochar (γ-Fe_2_O_3_@ BC) with banana peel as raw material by pot heating, used as a recoverable persulfate (PS) activator to degrade the organic pollutant bisphenol A (BPA) in water. Liu et al. [35] synthesized magnetic nitrogen-doped biochar (MNBC) from rice straw and degraded the herbicide metolachlor (MET) through a catalyst prepared by coupling it with peroxymonosulfate (PMS). It was found that easy separation and low toxicity were good performances of the catalyst. Shen et al. [36] proposed an economical and green method to prepare a graphite biochar catalyst for PS activation and explored the potential of sewage remediation. Magnetic graphitized biochar (GMBC) was prepared with a one-step method. The biochar derived from pine wood was used as a carrier, modified by potassium permanganate, and introduced as a sulfate activation to degrade stubborn sulfamethoxazole (SMZ). The catalytic efficiency was improved by electron transfer with 100% degradation within 60 min.

Bibliometrics is a method that utilizes mathematical and statistical techniques to describe, evaluate, and predict the current situation and development trends. By analyzing the amount of research articles, it provides an in-depth understanding of the scientific development in a specific field and determines future directions [37,38]. This paper collected 551 articles on magnetic biochar research in the Web of Science core collection database from 2011 to 2022. CiteSpace software (R 4.2.1) and RStudio (Version 3.1.4) were employed to visualize the publication trends, institutions, international collaborations, top 10 authors, and classic articles. The analysis examined the historical development, current status, and latest research trends of magnetic biochar in the field of wastewater treatment. Additionally, the paper summarized the pollutant removal mechanisms and modification methods of magnetic biochar. This study offers a thorough analysis and overview of magnetic biochar in the field of wastewater treatment, aiming to clarify the research direction in this field. The findings of this study can assist scholars in conducting further investigations and expedite the development of magnetic biochar.

## 2. Results and Discussion

### 2.1. Basic Characteristics of Publications

#### 2.1.1. Descriptive Analysis of Annual Publications

**Figure 1:**
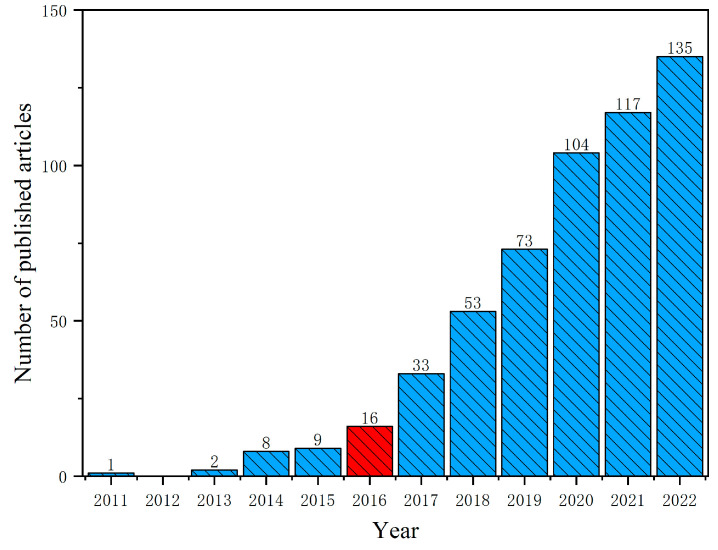
The number of publications in the field of magnetic biochar from 2011 to 2022. The year 2016 marked a turning point in the research of female biochar, as it experienced a significant and exponential growth from that year onwards.

The summary of annual publications describes the annual changes related to the specific topic of magnetic biochar, which can reveal research trends. According to Figure 1, the research on magnetic biochar started relatively late but developed rapidly. In 2011, there was a significant breakthrough in the field of magnetic biochar. Chen et al. [39] first proposed a new type of magnetic biochar to adsorb and remove pollutant phosphates in the water environment effectively. Three new magnetic biochar species (MOP250, MOP400, MOP700) were prepared by extracting biochar (orange peel powder) from discarded crops, which were chemically precipitated on Fe^3+^/Fe^2+^. After pyrolysis at different temperatures, MOP400 had the best adsorption capacity for organic pollutants, with the adsorption rate of up to 99.6 ± 0.0%, and MOP250 had the best adsorption capacity for phosphate, with the adsorption rate of up to 73.6 ± 0.1%; both were better than the non-magnetic biochar adsorption effect. The study highlights the potential of magnetic biochar as an effective adsorbent for removing organic pollutants and phosphate from water simultaneously. This emerging technology has laid a solid foundation for further exploration of magnetic biochar in the removal of pollutants from wastewater.

After a period of steady development from 2011 to 2016, the academic community has shown significant interest in researching magnetic biochar. As a result, the field has experienced rapid growth in the past three years, with a significant proportion (64.6%) of total published articles appearing during this time frame. This trend highlights the promising research potential of magnetic biochar as an innovative and effective adsorbent.

#### 2.1.2. Analysis of National Collaboration Networks

**Figure 2:**
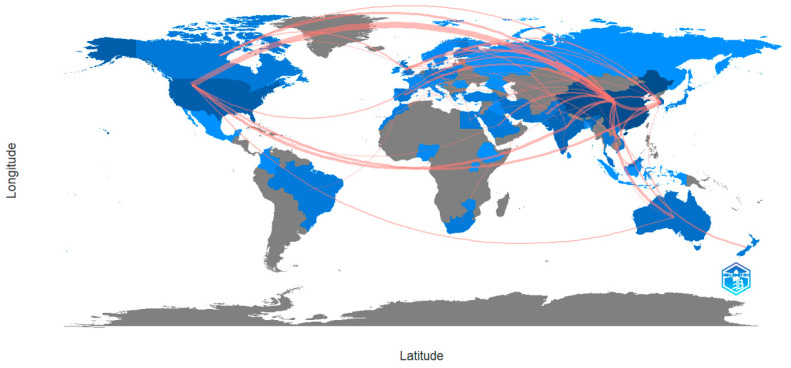
Geographical representation of collaborating countries. The darker the blue color in the picture, the more literature from countries/regions, and the closer the cooperation between countries/regions.

Figure 2 visually displays the research on national/regional magnetic biochar wastewater treatment networks and presents the communication and cooperation relationships between countries/regions. Overall, the cooperation between the United States in North America and China in Asia is the closest and most diverse regarding lines. Secondly, there is relatively close cooperation between North America and Oceania, as well as between North America and Republic of Korea.

According to Table 1, in terms of publication volume, China ranked first with 351 papers, indicating that China has conducted considerable research in this field and plays an essential role in theory and innovation. The United States was second with 37, and Republic of Korea ranked third with 25. China, the United States, and Republic of Korea are the most significant contributors to research output in this field. Additionally, the centrality is a statistical measure of the size of the connection in the knowledge graph network. It reflects the connection status of the nodes in the graph with other nodes and plays the role of linking the preceding and following nodes. The subjects with strong centrality not only possess the research value, but also play an important role in promoting the development of the field [37,40]. In study, China ranks first with a centrality of 0.637, reflecting its leadership in this field. After China, there are three countries with centrality values above 0.040: the USA (0.067), Republic of Korea (0.045), and India (0.044). Although some of these countries rank low in the number of articles, their scientific research achievements should not be ignored.

#### 2.1.3. The Influence of Institutions

This study employs three indicators, namely, frequency, centrality, and burst strength, to evaluate institutions’ overall impact in the magnetic biochar field. Table 2 shows that Chinese research institutions dominate the top 10 indicators, reinforcing China’s significant position in this field. Specifically, the Chinese Academy of Sciences stands out in terms of institution frequency and centrality, highlighting its preeminent position in the field of magnetic biochar. Notably, despite not ranking high in frequency and centrality, the University of Florida has the highest burst strength score of 3.26. A high burst strength indicates a surge in the citation of institutions’ achievements over a certain period, representing their academic influence. After conducting a thorough data analysis, it was revealed that Gao Bin’s team from the University of Florida had produced a series of impactful papers on the magnetic biochar method for removing heavy metals. These articles include two highly cited articles that have amassed over 400 citations [41,42]. Further details regarding the specifics of these articles will be discussed in Section 2.2.2.

#### 2.1.4. Cited Journals and Research Subjects

As shown in Table 3, the data were arranged in descending order by the h-index. The h-index was proposed by Jorge E. Hirsch in 2005 [43]. The h-index refers to the number of academic papers published by a scholar or journal, with N papers cited at least N times. Compared with the simple NP and total citation (TC) frequency, the h-index considers the quantity and quality of the academic output of researchers or journals. TC refers to the total citations of a journal’s paper on magnetic biochar in the field of wastewater treatment. NP refers to the number of publications in the field. PY_ Start refers to the year when the first literature in the field was published. This study uses the above four fundamental indicators to reveal the characteristics of literature sources in this field.

Considering the h-index, the literature published in Bioresource Technology from 2011 to 2022 had the most significant impact on the application of magnetic biochar in the field of wastewater, with 28 h-index, and 5774 total citations, far surpassing the second place. It also reveals the high impact of literature published in this journal. Considering the number of publications, Bioresource Technology has published 45 papers, followed by the Science of the Toal Environment (42 papers), and Chemsphere (33 papers). Overall, Bioresource Technology has a decisive influence and holds a prominent position in the application of magnetic biochar in the field of wastewater.

**Figure 3:**
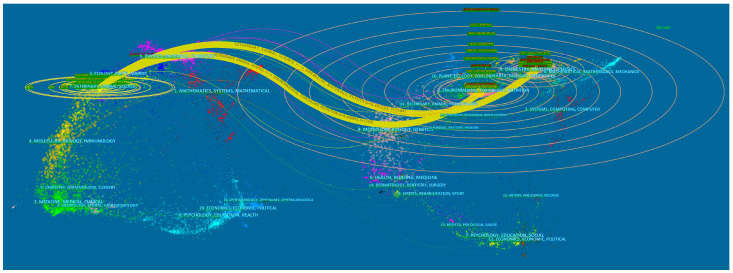
Disciplinary double-map overlay for magnetic biochar. The citing journal is presented on the left, while the corresponding cited journal is shown on the right. The thickness of the yellow curve in the middle reflects the extent of citation links between different disciplines, thereby indicating their proximity.

Discipline correlation analysis is a crucial step in correlating cited literature with the appropriate discipline. This process not only facilitates the identification of the development trajectory of a discipline but also enables scholars to comprehensively comprehend the citation patterns [44]. In this study, we employed CiteSpace to demonstrate a disciplinary double-map overlay for applying magnetic biochar in wastewater treatment. Figure 3 illustrates the citing journal on the left and the corresponding cited journal on the right. The thickness of the yellow curve in the middle indicates the proximity of citation links between disciplines. It is evident that there are two citation pathways for research on magnetic biochar. The citing journals predominantly address discipline in “veterinary, animal science”. On the other hand, the cited journals originate from two disciplines: “chemistry, materials, physics” (z: 3.267103) and “environmental, toxicology, nutrition” (z: 4.499892). Notably, the literature on applying magnetic biochar in wastewater treatment has been mainly published in “environmental, toxicology, nutrition” journals.

### 2.2. Analysis of Influential Authors and Articles

The basic characteristics of publications can help researchers gain a preliminary understanding of the overall situation of magnetic biochar, but the analysis of highly cited articles and authors can help researchers more accurately and effectively identify research achievements with solid academic influence.

#### 2.2.1. Analysis of Top 10 Authors

RStudio was used to analyze the researchers’ publishing articles in the field of magnetic biochar. The larger the circle area, the higher the citation frequency of the author and the more important the academic influence. Gao is one of the primary scholars in the early period, with his work laying a theoretical foundation for subsequent research. In second place was Pittman, and in third place was Mohan.

**Figure 4:**
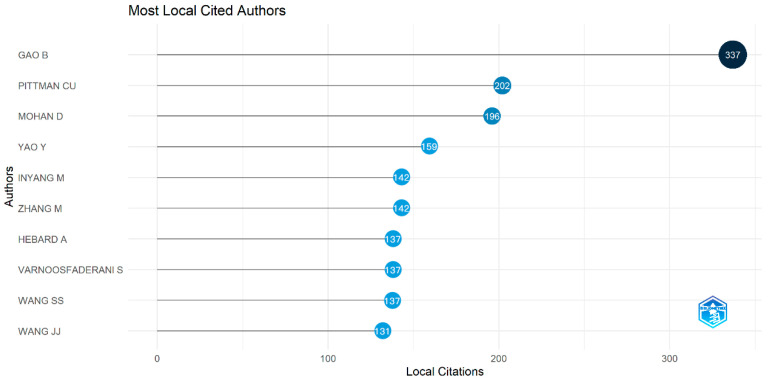
Top 10 local cited authors. “Local citations” refers to the number of times an article has been cited by other papers in the same database in this study.

Figure 4 analyzes the top 10 researchers in the field of magnetic biochar. The larger the circle area, the higher the citation frequency of the author and the more important the academic influence. Bin Gao is the most influential researcher in this field. His research team has conducted extensive research on the removal of arsenic by magnetic biochar, with two articles cited more than 400 times. In 2013, Gao et al. [41] employed a magnetic biochar adsorbent that was prepared via the pyrolysis of biomass treated with ferric chloride. The Langmuir maximum adsorption capacity for As(V) was 3147 mg/kg, comparable to that of many commercial adsorbents. Thus, the magnetic biochar adsorbent can potentially be used to remove arsenic during water treatment efficiently. Further investigation into the magnetism of the adsorbent revealed a coercivity field of 34.1 Oe and a saturation magnetization of 69.2 emu/g, which is comparable to the magnetism of pure γ-Fe_2_O_3_ material (76.0 emu/g). It is advantageous for the easy recovery of the biochar/γ-Fe_2_O_3_ adsorbent to deal with pollutants after use. Overall, the adsorbent exhibited a strong adsorption capacity for arsenic. Two years later, Gao et al. [42] successfully developed magnetic biochar by pyrolyzing a mixture of synthetic pine wood and natural hematite. The biochar could be easily removed from the external magnet due to its magnetic properties. The maximum Langmuir adsorption capacity of PB (biochar without hematite modification) for As was 265 mg/kg. The γ-Fe_2_O_3_ particles on the carbon surface served as adsorption sites through electrostatic interaction, thereby significantly increasing the maximum Langmuir adsorption capacity of HPB (biochar modified with hematite) to 429 mg/kg. Although the adsorption capacity of this new magnetic biochar is not as good as that reported two years ago, it is comparable to the sorption capacity of magnetic biochar derived from chemically-treated agricultural minerals [39]. Furthermore, the low-cost and abundant source of biomass and hematite minerals make them ideal for widespread usage.

Secondly, Pittman Cu and Mohan D were cited over 300 times, with three articles co-authored with citation frequencies of 354, 143, and 126. In 2014, they produced rapidly pyrolyzed magnetic oak wood biochar (MOWBC) and magnetic oak bark biochar (MOBBC) to remediate cadmium and lead contamination [29]. Batch adsorption experiments were conducted under varying conditions, including temperatures of 25–45 °C, pH values of 2–7, and different solid–liquid ratios. The highest lead and cadmium removal rates were found at pH 4–5. The saturation magnetization of MOBBC and MOWBC was 4.47 and 8.87 emu/g, respectively. Subsequently, they dispersed α-Fe_2_O_3_ and Fe_3_O_4_ in Douglas fir biochar (BC) with a high specific surface area (663 m^2^/g) [45], which was used for the rapid removal of nitrate and fluoride ions from water using magnetic separation. The magnetic biochar exhibited the best adsorption effect on nitrate and fluoride at pH 2–10, and the adsorption behavior was further explored at temperatures of 298–318 K through the Langmuir and Freundlich isotherm models. It was found that magnetic biochar had a higher Langmuir adsorption capacity of 15 mg/g for nitrate and 9 mg/g for fluoride as compared to other biochar and iron oxide adsorbents. In 2018, the authors collaborated once again to synthesize magnetic biochar (MBC) by precipitating magnetite (Fe_3_O_4_) onto Douglas fir biochar (NBC) via wet rapid pyrolysis [46]. The resulting MBC exhibited excellent efficiency in removing Pb^2+^ and Cd^2+^ from wastewater. At a pH of 5 and temperature of 45 °C, the maximum Langmuir adsorption capacity of NBC for Pb^2+^ and Cd^2+^ was found to be 40 and 16 mg/g, respectively, while that of MBC was 27 and 11 mg/g, respectively. The multiple partnerships between the two researchers have provided evidence that magnetic biochar exhibits efficacy not only in removing heavy metals, such as cadmium and lead, but also in generating commendable adsorption capacity for inorganic compounds, including nitrate and fluorine. Its exceptional environmental adsorption properties render magnetic biochar a highly promising adsorbent with enormous potential in the current scenario.

Most of the authors ranking in the top ten have conducted research on magnetic biochar’s adsorption performance. However, it is worth noting that Zeng, who is not depicted in Figure 4, has 278 citations. Notably, four highly cited articles have been published on using magnetic biochar in catalysis, making exceptional contributions to the field of magnetic biochar catalysis. Zeng et al. [47] investigated the preparation of a silver nanoparticle supported on polydopamine magnetic biochar (MC-PDA Ag) catalyst through the method of in situ reduction. The researchers characterized the catalyst (MC-PDA Ag) and found that it exhibits excellent catalytic performance for dyes (MB, RhB and MO) in the presence of NaBH_4_. The reduction efficiency of the catalyst to MB in five minutes was found to be over 90% under varying pH (3–11) and different concentrations of NaNO_3_ (0–0.5 M), demonstrating the potential universal performance of the catalyst in dye wastewater. In addition, the capacity of the catalyst did not decline after five cycles, and it could also be separated by an external magnet, indicating the catalyst’s reusability and easy separation. These findings suggest that the composite catalyst has great application potential in treating dye wastewater. Subsequently, Zeng et al. [48] loaded MnFe_2_O_4_ onto biochar, successfully preparing a new magnetic biochar composite to remove tetracycline (TC). The 1:2 complex, used as the heterogeneous photocatalyst, demonstrated a tetracycline degradation rate of 95% in the presence of 100 mmol L^−1^ H_2_O_2_ under natural pH 5.5, irradiating a 40 mg L^−1^ solution with visible light for 2 h. These findings indicate that in addition to the high removal efficiency of adsorption in the field of magnetic biochar, the degradation efficiency of catalysis in the field of magnetic biochar is also excellent. Moreover, the catalyst itself possesses the functions of recycling and easy separation.

#### 2.2.2. Analysis of Top 10 Articles

The application of magnetic biochar in the field of sewage treatment has garnered a significant amount of novel research articles, and identifying the most influential ones can aid researchers in gaining a quick understanding of the current forefront and hotspots of the field. Table 4 analyzes the top 10 most highly cited magnetic biochar articles, organized by RStudio, along with key information such as title, author, target pollutants, total citation (TC), year and sources. Generally, most articles with high citation were published between 2011 and 2019, with the Bioresource Technology journal being the primary source.

As shown in Table 4, the top 10 classic literatures focus primarily on applying magnetic biochar to remove organic pollutants and heavy metals. Specifically, the research on organic pollutants can be summarized as follows. Chen et al.’s [39] article “A novel magnetic biochar effectively sorbs organic pollutions and phosphate” published in 2011 was cited the most, reaching 652 times, ranking first; this paper was the first time that magnetic biochar was prepared by adding magnetism to biochar to remove organic pollutants and phosphates in wastewater. Three kinds of magnetic biochar (MOP250, MOP400, MOP700) were prepared by using a chemical coprecipitation method using orange peel as raw material, magnetite as magnetic target medium, and pyrolysis at different temperatures. The experiment shows that MOP400 has the highest adsorption capacity for organic pollutants, with the adsorption rate of up to 99.6 ± 0.0%, and MOP250 has the highest adsorption capacity for phosphate, with the adsorption rate of up to 73.6 ± 0.1%. This kind of magnetic biological carbon is used to remove organic pollutants and phosphate in wastewater simultaneously, and the effect is excellent. Shan et al. [54] utilized the ball-milling method to prepare ultrafine magnetic biochar/Fe_3_O_4_ with exceptional adsorption capacity for drug compounds. The maximum adsorption capacities of carbamazepine (CBZ) and tetracycline (TC) were 62.7 mg/g and 94.2 mg/g, respectively. This study is noteworthy as it introduces mechanochemical degradation technology (ball milling) after the physical adsorption step, for the complete degradation of organic pollutants. Unlike conventional oxidation techniques, milling exploits mechanical energy to activate chemical reactions and has been demonstrated to treat waste, including organic pollutions and pharmaceuticals [55,56]. Results revealed that after 3 h of the ball-milling treatment on adsorbents that have previously adsorbed pollutants, the degradation rate of adsorbed TC exceeded 99%, while approximately half of CBZ remained. However, the addition of 300 mg SiO_2_/g milling agents resulted in a significant increase in the degradation effect of CBZ to 98.4%. This intriguing technological coupling indicates that ball milling is not only an economical approach for the preparation of efficient magnetic adsorbents, but also an effective method for degrading pollutants adsorbed on waste adsorbents, thereby reducing their environmental risks.

The research on heavy metals is as follows. Zhang et al.’s [41] article “Preparation and characterization of a novel magnetic biochar for arsenic removal” published in 2013 was cited 450 times, ranking second. This paper mainly studies the preparation of a magnetic biochar γ-Fe_2_O_3_ composite by pyrolysis of biomass treated with FeCl_3_. Through characterization and analysis, it is found that this magnetic biochar has excellent ferromagnetic properties, with a saturation magnetization of 69.2 emu/g, and the maximum adsorption capacity of γ-Fe_2_O_3_ particles on arsenic in the composite material is about 4237 mg/kg, which is similar to the maximum adsorption capacity of pure γ-Fe_2_O_3_ particles (4643 mg/kg). The results show that the magnetic biochar can easily separate the metal arsenic from the solution through the magnetic properties. Mohan et al. [29] synthesized magnetic oak wood biochar (MOWBC) and magnetic oak bark biochar (MOBBC) through rapid pyrolysis of oak wood and oak bark as raw materials. Their study revealed that MOBBC and MOWBC exhibited saturation magnetization values of 4.47 and 8.87 emu/g, respectively. Under conditions of pH = 5.0 and a temperature of 25 °C, magnetic biochar dosed at 10 g/L achieved nearly 100% removal of lead and removal rates for cadmium ranging from 53% to 99%. These results demonstrate the effectiveness of magnetic biochar in removing heavy metals such as lead and cadmium from water. In a study by Sun et al. [49], biochar derived from wood waste was combined with an aqueous solution of FeCl_3_. Through bridging CeO bonds and cation-π interactions, the resulting Fe-BC composite material effectively removes potential toxic elements, inherent cations, and chlorinated organics found in hydraulic fracturing wastewater (FWW).

#### 2.2.3. Cluster of Keywords’ Occurrence

Keyword analysis can identify the frontier hot issues in a research cycle. Using RStudio, high-frequency keywords were extracted from the literature, and clustering analysis was performed. In this way, the correlation and timeline relationship between keywords can be displayed, reflecting trends of research ideas. The keywords of an article represent the main ideas of the research. Keyword prominence refers to the rapid increase in the frequency of a keyword within a certain period, helping to track research hotspots and new trends in a certain period.

**Figure 5:**
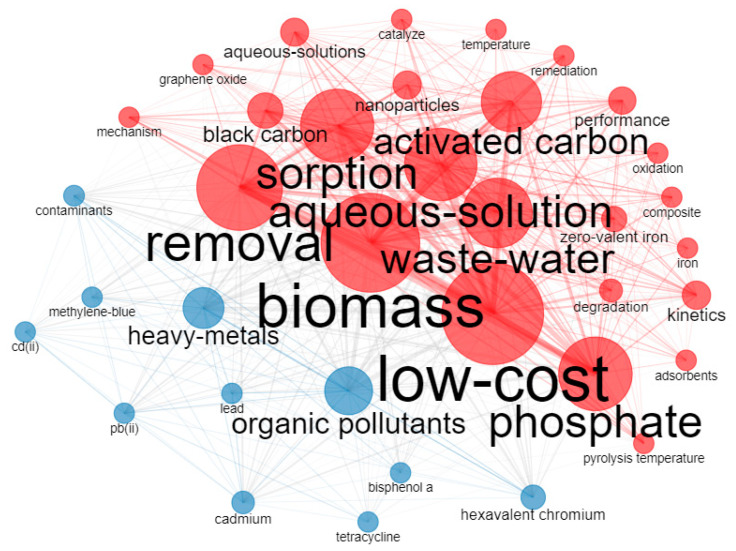
Overlay visualization map of keywords co-occurrence network.

With visual analysis via RStudio, the keyword clusters are shown in Figure 5. The density of the clusters indicates the degree of connection within the overall topic, with a higher intensity corresponding to greater topic maturity. In addition, the larger the font size and the darker the color of the text in the circle, the more times the keyword is repeated, and the broader the content involved. The keywords with the boldest font in the dataset are removal, biomass, waste-water, low-cost, phosphate, aqueous-solution, sorption, activated carbon, heavy metals, and organic pollutants.

The clusters’ analysis reveals keywords’ evolution trend from 2011 to 2022. An in-depth visual analysis of the most mentioned keywords shows that these two colors can show different research hotspots. The primary objective of the red circle is to investigate the various methods of preparing Biochar and improve its removal mechanisms. This involves exploring biomass selection, cost-effective approaches, temperature optimization, pyrolysis techniques, black carbon formation, magnetic biochar synthesis, water treatment, adsorption mechanisms, degradation processes, and removal efficiency. On the other hand, the blue circles mainly focus on the removal of different pollutants, such as arsenic (As), Cd(II), Pb(II), heavy metals, tetracycline, methylene blue, etc.

#### 2.2.4. Keyword Burst Analysis

**Figure 6:**
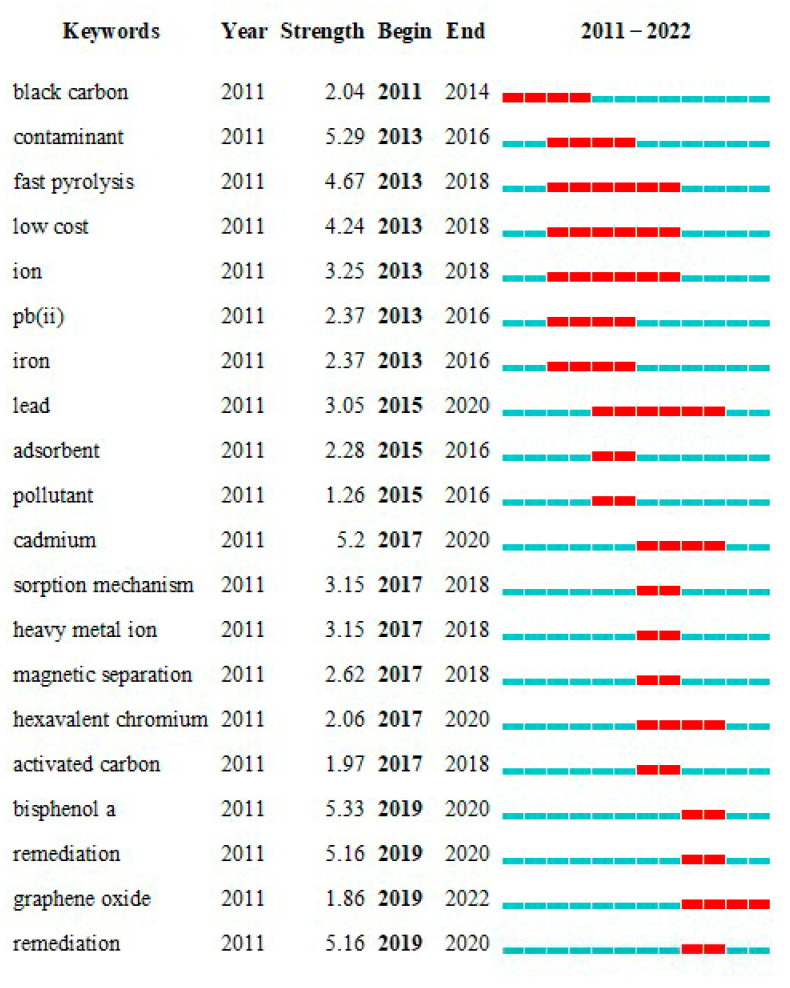
Keywords with the strongest citation bursts. The keyword burst intensity is denoted by “strength,” while the duration is indicated by “begin” and “end”. The red square in the figure depicts the time span from the beginning to the end of the surge, with each grid representing one year. The keywords are presented in chronological order.

By analyzing keyword burst intensity, researchers can detect and track hot topics and research trends and understand the changes in current topics [57]. As shown in Figure 6, the highly emphasized keywords, namely “black carbon,” “continuous,” and “fast pyrolysis,” emerged between 2011 and 2018, primarily aimed at tracing the evolutionary trajectory of biochar. The production of biochar was found to exhibit proficient pollutant removal capabilities, thereby laying the groundwork for investigating modified biochar. Following highly emphasized keywords included “low cost,” “iron,” and “pb (ii),” whereby researchers concentrated on identifying eco-friendly and cost-effective raw materials to manufacture biochar [58], embedded with magnetism through ions, for the removal of lead-heavy metals from water [59]. From 2015 to 2016, the keywords with high frequency were “absorbent” and “pollutant,” and “lead” can also be included during this time frame. The main research direction was investigating the adsorption [60] and catalytic [61] functions of magnetic biochar and analyzing its effectiveness in removing pollutants. The dominant factor was the adsorption capacity, with heavy metal leads [62] being a key research hotspot. From 2017 to 2020, keywords such as “cadmium”, “sorption mechanism”, “heavy metal ion”, “magnetic separation”, and “hexavalent chromium” have been highlighted. The research predominantly focused on purifying water sources contaminated with Cd, Cr heavy metal ions pollution. The absorption mechanism was analyzed, and effective biochar raw materials were explored.

The highlighted time period from 2019 to 2022 stands out as particularly significant. During this period, keywords such as “bisphenol A,” “remediation,” and “graphene oxide” are prominently displayed. These emerging keywords provide valuable insights into the research directions pursued by scholars and the innovative discoveries they have made. Heo et al. [63] utilized a one-pot hydrothermal method to prepare a novel magnetic biochar composite material embedded with CuZnFe_2_O_4_ (czf-biochar). This composite material possesses remarkable properties such as fast kinetics, high adsorption performance, easy magnetic separation, and the ability to be repeatedly used. As a result, it demonstrates excellent efficacy in eliminating bisphenol A (BPA) and sulfamethazole (SMX) from water. Zhai et al. [64] prepared a novel Z-scheme magnetic biochar@CoFe_2_O_4_/Ag_3_PO_4_ photocatalyst that utilizes visible light (λ ≥ 420 nm). Under this irradiation, bisphenol A was effectively degraded, with photocatalytic and mineralization efficiencies reaching their peak within 1 h at 91.12% and 80.23%, respectively. Mahmoud et al. [65] developed a novel biosorbent, nitrogen-doped Graphite oxide hydrogel shrimp shell magnetic biochar. The biosorbent exhibited a remarkable adsorption capacity of 350.42 mg/g for Cr(VI) when the initial concentration was 100 mg/L, the contact time was 180 min, and the pH was adjusted to 1. Furthermore, the biosorbent demonstrated excellent performance in removing Cr(VI) from different water sources. In tap water, seawater, and wastewater, the removal rates reached 99.79%, 99.20%, and 98.00%, respectively. These findings highlight the broad applicability of the developed biosorbent. During this period, research efforts have primarily focused on removing organic pollutants from water sources. Scholars have increasingly recognized the importance of catalytic research and have started to explore the combination of adsorption and catalysis to address various types of water pollution.

## 3. Removal Mechanisms of Magnetic Biochar

Summarizing and analyzing the removal mechanisms of pollutants with magnetic biochar is important for efficiently using magnetic biochar in the remediation of water environments. This paper summarizes the main adsorption mechanisms and briefly discusses the relevant literature based on the previous bibliometrics study.

### 3.1. Adsorption Mechanisms of Heavy Metal

The electrostatic attractions of functional groups play a significant role in the mechanism of Cd(II) removal by MBC, with iron oxides playing a pivotal role in this process [66]. For example, Zhang et al. [67] prepared a novel biochar-based magnetic nanocomposite (GSMB) from white tea waste via a green synthesis method, using ferric chloride as raw materials on the biochar with pyrolysis at 300 °C. It revealed that strong electrostatic attraction between the negatively charged functional groups such as -COO- and -OH- on the surface of GSMB and the positively charged Cd(II) is the primary adsorption mechanisms. And the high affinity of iron oxides to Cd(II) play an important role during Cd(II) adsorption by GSMB.

Ion exchange is mainly the replacement between Fe^3+^/Fe^2+^ ions or alkali metal ions contained in magnetic biochar and heavy metal ions [68]. Phoungthong et al. [69] prepared magnetic biochar (MBS) from sewage sludge by controlling the pyrolysis temperature at 400–500 °C to remove Al^3+^ and Cu^2+^ ions in wastewater effectively. The minerals present in MBS, such as K^+^ and Zn^2+^, are key factors that play a significant role in the exchange process. The leaching experiments found that the release of K and Zn can influence the adsorption of Al^3+^ and Cu^2+^. Zhang et al. [70] successfully synthesized a bifunctional MBC by combining EDTA and chitosan, aiming to simultaneously remove Methyl orange, Cd(II) and Zn(II). The experimental findings indicated that the ion exchange process between Fe(II) and Cd(II)/Zn(II) resulted in the decrease of Fe(II) content within Fe_3_O_4_.

Redox interaction utilizes Fe^0^ [71], FeO [72], redox-active functional groups and other reducing substances [73] loaded on MBC to alter the valence state of heavy metals, thereby reducing their toxicity and mobility. Yin et al. [24] prepared MBC through K_2_FeO_4_-promoted oxidative pyrolysis of pomelo peel for adsorbing Cr(VI). The FTIR spectra demonstrated that the C-O groups of MBC notably increased after the adsorption of Cr(VI), indicating that the reduction of Cr(VI) to Cr(III) occurred during this process. Lyu et al. [74] used a composite material of iron-modified bamboo biochar and pre-magnetized CaMgAl layered double hydroxides (Fe-BC@LDH). It is noteworthy that the formation of ternary FeOCdAs bonding configuration, redox conversion of As(III) to As(V), intra-sphere complexation of MOAs/Cd (MFe, Ca, Mg, Al), electrostatic attraction, and coprecipitation of mica and hydroxy iron-cadmium are beneficial for the removal of As(III) and Cd(II) in the binary competitive system.

As for surface complexation, it refers to the formation of complexes through interactions between an electron donor and an electron acceptor. An electron donor is responsible for providing an electron pair, while the electron acceptor is typically composed of metal ions or organic compounds [68]. In fact, the adsorption mechanism of magnetic biochar for heavy metals is often complex and cannot be explained by a single mechanism. Qu et al. [75] conducted a study on the removal of heavy metal Cr(VI) from polluted water using magnetic biochar doped with Polyethylenimine (PEI) grafted nitrogen (N) (PEIMW@MNBCBM). Various mechanisms were involved in the adsorption process, including electrostatic attraction, surface complexation, precipitation, reduction, and pore filling. Another study by Wang et al. [76] involved the preparation of la-doped magnetic biochar using the coprecipitation method to remove Sb(V) from wastewater. This study found that other mechanisms, such as hydrogen bonding, electrostatic attraction, and ligand exchange, were also involved in Sb(V) removal.

### 3.2. Adsorption Mechanisms of Organic Compound

The adsorption mechanism of magnetic biochar for organic compounds, another major category of pollutants, mainly involves several processes such as hydrogen bonding, electrostatic interactions, pore filling, and π-π stacking interaction.

Pore filling is commonly recognized as a fundamental mechanism for the removal of pollutants by adsorbents. It refers to the process in which the adsorbent enters the pores of the adsorbent material [77]. Consequently, researchers consistently devote significant efforts to enhancing the specific surface area and porosity of magnetic biochar to improve the removal efficiency of organic pollutants [78]. Dou et al. [79] introduced KOH-activated fish scale biochar for enhanced ciprofloxacin adsorption and attributed the observed increase to the pore-filling effect. In a study conducted by Fan et al. [80], they investigated the adsorption effects of modifying chitosan when both tetracycline and Cu^2+^ were simultaneously removed using magnetic porous biochar. The results showed that the modification had contrasting effects on the adsorption of Cu^2+^ and TC. Specifically, the adsorption amount of Cu^2+^ increased from 17.32 mg g^−1^ to 65.87 mg g^−1^ after modification. This enhancement was attributed to the complexation and electrostatic interaction between Cu^2+^ and the N/O functional groups introduced by the chitosan modification. On the other hand, the chitosan modification weakens the pore filling, resulting in a significant decrease in the absorption capacity of TC from 471.77 mg g^−1^ to 241.33 mg g^−1^. This finding further confirms the crucial role played by pore-filling effects in the adsorption capacity of magnetic biochar for organic pollutants.

Electrostatic adsorption refers to the interaction between the adsorbate and the adsorbent where their opposite charges attract each other. Magnetic biochar, due to its ionizable functional groups on the surface, can acquire a surface charge that enables it to attract and adsorb organic pollutants with opposite electrical properties in water through electrostatic attraction. This effect is commonly found to be the primary mechanism involved in the adsorption process of magnetic biochar for organic dyes pollutants [81]. When the surface charge of magnetic biochar is negative, it can effectively remove Acid Blue 158 [82] and malachite green [83] through electrostatic adsorption. Therefore, the strength of electrostatic adsorption can be regulated by manipulating the pH value of the solution [84].

The hydrogen bond is a phenomenon that occurs when the hydrogen atoms of functional groups on the surface of magnetic carbon interact with atoms that have a high electronegativity and small radius. This interaction involves the formation of covalent bonds, and it is considered to be a potential mechanism by which magnetic biochar can adsorb organic molecules [85]. The pyrolysis process can additionally amplify the aromaticity of magnetic carbon, resulting in an increased presence of functional groups on its surface [16]. These functional groups are capable of engaging in a π-π stacking interaction with organic molecules, thereby facilitating their adsorption and removal of pollutants. π-π stacking interaction and hydrogen bonding often appear together as the main mechanism in the removal of organic pollutants [86]. Chen et al. [87] developed a new β-cyclodextrin (β-CD) modified magnetic alginate/biochar (β-CD@MBCP) material for the co-adsorption of methylene blue (MB) and lead. Results showed the uptake of MB was facilitated by π-π stacking between the C = C bonds of MB and BC, and the characteristic peak intensity of the O-containing functional groups on β-CD@MBCP decreased after adsorption of MB, indicating the formation of hydrogen bonds between MB and the –OH or –COOH groups on the surfaces of the β-CD@MBCP during MB uptake. Zhao et al. [88] also discovered in their experiments that the primary adsorption mechanisms for enhancing the adsorption of fluoroquinolones (FQs) were the π-π stacking interaction and hydrogen bonding.

## 4. Modification Methods of Magnetic Biochar

The surface area and porosity of biochar formed after simple pyrolysis treatment of biomass are typically low, resulting in poor adsorption performance. Conversely, magnetic biochar (MBC) has the advantage of being magnetic and easy to recover and reuse. However, the addition of magnetic material can block the pores, leading to lower adsorption performance. To overcome this issue, researchers commonly employ different modification methods to reshape the physicochemical properties of MBC in order to achieve better removal efficiency. Table 5 provides a summary of several standard modification methods and their respective advantages in terms of their effects on removal.

## 5. Existing Problems

There has been much research on the application of magnetic biochar in sewage treatment. While these studies have contributed to the theoretical and practical understanding of magnetic biochar, several issues still need to be addressed. In future research and development, researchers must shift their focus toward the following aspects.

- Environmental protection: Currently, it has been found that the leaching of metals, such as Fe, in magnetized biochar has minimal impact on the surrounding environment, and the toxicity is relatively safe. However, some metals, such as Mn, Co, and Ni, may cause particular damage to the environment through leaching. In addition, it is vital to study magnetic biochar’s transformation and potential toxicity in long-term environmental applications. Although the stability of magnetic biochar is reliable, its catalytic properties gradually diminish with repeated cycles of use. During the degradation of organic pollutants, magnetic biochar may undergo incomplete mineralization, resulting in degradation by-products that are more toxic than the initial pollutants. However, there is limited research on the toxicity of degradation by-products, suggesting more attention is needed to transform and apply magnetic biochar in environmental remediation.
- Economy: Biochar is derived from low-cost biological waste. However, the addition of metal ions in magnetic biochar, which enhances its magnetic properties and degradation effectiveness, often leads to increased costs. Unfortunately, the preparation costs of magnetic biochar have not received sufficient attention, impeding its practical application. Therefore, it is crucial to consider the transportation and pre-processing costs for bulk-scale operations.
- Novelty: The current compilation of articles primarily addresses the elimination of heavy metals and organic pollutants, with limited consideration for inorganic pollutants. Additionally, the emphasis is predominantly on adsorption as the removal mechanism. To fully achieve mineralization and pollutant removal, it is necessary to integrate multiple technological approaches, such as adsorption catalysis.

## 6. Materials and Methods

### 6.1. Data Sources

**Figure 7:**
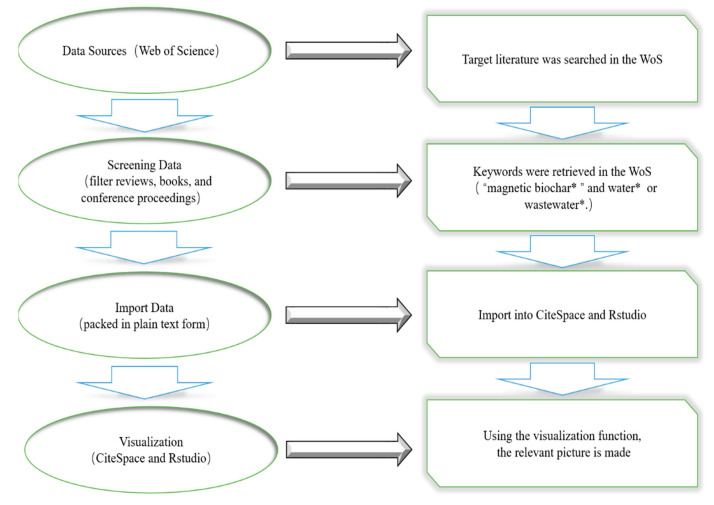
The overall retrieval procedure. In the Web of Science algorithm, the use of double quotation marks represents the whole word, and the asterisk (*) represents various word tenses.

Web of Science (WoS) is an academic information integration platform belonging to Clarivate. The core collection of WoS is an essential database for obtaining global academic information, which includes multiple authoritative and influential academic journals around the world, covering a wide range of fields. For this study, the Web of Science (WoS) core collection database was searched on 26 January 2023. To find articles related to magnetic biochar materials, the search term was “magnetic biochar*” and water* or wastewater*”. After filtering by excluding reviews, books, and conference proceedings, etc., a total of 551 articles published between 2011 and 2022 were retrieved in plain text file format. The exported data were then transferred to CiteSpace and RStudio to draw relevant maps for bibliometric analysis. The overall retrieval procedure is shown in Figure 7.

### 6.2. Mapping Tools

Bibliometric is a method used to analyze a large number of academic studies, summarize experiences, and explore hot spots and innovations in the field [93,94]. Commonly used bibliometrics tools include CiteSpace, RStudio, VOSviewer, Bibliometrics, etc. [95]. In this study, CiteSpace software (R 4.2.1) provides the basic characteristics of the publication information retrieved from WoS. The software identified the top 10 countries and top 10 institutions that are significant in the research of magnetic biochar in wastewater treatment. By examining centrality and frequency parameters, it can clearly understand the contributions made by various countries and institutions in this field. Moreover, CiteSpace software also offers disciplinary double-map overlay and keyword burst analysis to reveal the development trajectory of the subject, track the hot topics and research trends. In order to facilitate a more intuitive and comprehensive analysis of articles, we utilized RStudio software package (Version 3.1.4) to develop national collaboration networks, as well as the top 10 journals, authors, articles, and keywords within the dataset. Additionally, high-frequency keywords were extracted from the literature, and cluster of keywords co-occurrence was performed.

## 7. Conclusions

This study conducts a bibliometric analysis of 551 articles published in the Web of Science (WOS) core collection from 2011 to 2022, using the CiteSpace and RStudio software. In 2011, magnetic biochar was first applied in wastewater treatment and showed a promising effect. Subsequently, scholars’ attention to magnetic biochar has continuously increased, resulting in a growing number of published papers. High-frequency keyword clustering revealed that scholars’ research on magnetic biochar mainly focused on two aspects: the preparation methods of biochar and the removal mechanisms for various pollutants. Based on the top 10 authors and classic literature, it is discovered that magnetic biochar mainly targets heavy metals and organic pollutants. Among all the research, the frequency of publications from the Chinese Academy of Sciences is the highest, and cooperation between China and the United States is the most prevalent. More high-quality articles in this field are published in the journal Bioresource Technology. From the h-index, the team led by Gao from the University of Florida is the most influential. Their discovery of a low-cost method for producing magnetic biochar for the removal of heavy metal Pb^2+^ has promoted the development of the magnetic biochar field. Based on the summary of the keywords with the strongest citation bursts, it has been found that the removal of organic pollutants by magnetic biochar has become a hotspot in recent years. Lastly, based on bibliometric statistics, a brief summary of different modification methods and removal mechanisms of magnetic biochar is presented, and several future suggestions are proposed to address the existing shortcomings of magnetic biochar.

- Environmental Protection: The focus should be on exploring the leaching behavior of metal ions, and promptly discovering their impact on the surrounding environment. For large-scale applications, it is necessary to increase the recycling of magnetic biocarbon waste and avoid causing secondary pollution as much as possible. When degrading organic pollutants, toxic by-products (such as aromatic compounds) should be controlled and eliminated.
- Economic Efficiency: Steps and energy consumption in the pre-processing stage should be minimized as much as possible. It is preferred to avoid using methods that involve doping with precious metals for modification. Since nitrogen doping can enhance adsorption performance by providing more surface functional groups, it is worth considering the direct preparation of nitrogen-rich magnetic biochar using nitrogen-rich food processing waste such as soy sauce residue and vinegar residue, without introducing external nitrogen sources.
- Joint Technology: Exploring the adsorption–catalytic coupling of magnetic biochar to achieve rapid and complete mineralization of organic pollutants is suggested, as is investigating the simultaneous removal of various types of pollutants, including inorganic pollutants. Lastly, applying different modification methods to enhance magnetic biochar’s adsorption and degradation capabilities is recommended.

## Figures and Tables

**Table 1:** Top 10 countries by number of publications.

No.	Country	Centrality	Articles
1	People’s Republic of China	0.637	351
2	United States of America	0.067	37
3	Republic of Korea	0.045	25
4	India	0.044	24
5	Iran	0.022	12
6	Malaysia	0.018	10
7	Brazil	0.015	8
8	Australia	0.013	7
9	Czech Republic	0.011	6
10	Thailand	0.011	6

**Table 2:** Ranking of publications of top 10 institutions in the field of magnetic biochar. Notes: Chinese Academy of Sciences in this study is a conglomerate of many institutes/units.

No.	Institutions (Frequency)		Institutions (Centrality)		Institutions (Burst Strength)	
1	Chinese Academy of Sciences	22	Chinese Academy of Sciences	0.15	Chinese Academy of Sciences	1.32
2	Hunan University	16	Guangzhou University	0.12	Hunan University	2.75
3	Chinese Academy of Agricultural Sciences	11	Chinese Academy of Agricultural Sciences	0.08	Chinese Academy of Agricultural Sciences	1.52
4	University of Florida	10	University of Florida	0.08	University of Florida	3.26
5	Zhejiang University	10	Hunan University	0.04	Ministry of agriculture and rural affairs	2.01
6	Northwest A&F University	10	Jawaharlal Nehru University	0.03	Curtin University	2.67
7	Central South University	10	Zhejiang University	0.02	Central South University	3.2
8	Guangzhou University	9	Northwest A&F University	0.01	Shantou University	1.54
9	Jawaharlal Nehru University	9	Mississippi State University	0.01	Jawaharlal Nehru University	2.34
10	Mississippi State University	8	Central South University	0.01	Mississippi State University	3.08

**Table 3:** Ranking of top 10 publications in the magnetic biochar field.

No.	Journal	h-Index	TC	NP	PY_ Start
1	Bioresource Technology	28	5774	45	2011
2	Science of the Total Environment	25	2234	42	2016
3	Chemsphere	21	1883	33	2016
4	Journal of Hazardous Materials	17	1254	22	2016
5	Chemical Engineering Journal	15	1498	19	2014
6	Environmental Science and Pollution Research	14	820	24	2015
7	Journal of Environmental Management	12	440	17	2017
8	Journal of Environmental Chemical Engineering	10	280	20	2017
9	Rsc Advances	10	580	12	2015
10	Journal of Cleaner Production	9	526	14	2017

**Table 4:** Top 10 highly cited publications about the magnetic biochar being used for water treatment research.

No.	Title	Authors	Target Pollutants	TC	Year	Sources
1	A novel magnetic biochar efficiently sorbs organic pollutants and phosphate	Chen, BL	Naphthalene, p-nitrotoluene and phosphate	652	2011	Bioresource Technology [39]
2	Preparation and characterization of a novel magnetic biochar for arsenic removal	Zhang, M	As(V)	450	2013	Bioresource Technology [41]
3	Removal of arsenic by magnetic biochar prepared from pinewood and natural hematite	Wang, SS	As(V)	415	2015	Bioresource Technology [42]
4	Cadmium and lead remediation using magnetic oak wood and oak bark fast pyrolysis bio-chars	Mohan, D	Pb^2+^ and Cd^2+^	354	2014	Chemical Engineering Journal [29]
5	Multifunctional iron-biochar composites for the removal of potentially toxic elements, inherent cations, and hetero-chloride from hydraulic fracturing wastewater	Sun, YQ	potentially toxic elements (Cu(ii), Cr(VI), Zn(ii) and As(V)), inherent cations(Na, Ca, K, Mg, Sr and Ba), and 1,1,2-TCA, and TOC	288	2019	Environment International [49]
6	Adsorption of Cd(II) from aqueous solutions by rape straw biochar derived from different modification processes	Li, B	Cd(II)	275	2017	Chemsphere [50]
7	Adsorption kinetics of magnetic biochar derived from peanut hull on removal of Cr(VI) from aqueous solution: Effects of production conditions and particle size	Han, YT	Cr(VI)	275	2016	Chemsphere [51]
8	Sustainable efficient adsorbent: Alkali-acid modified magnetic biochar derived from sewage sludge for aqueous organic contaminant removal	Tang, L	tetracycline	269	2018	Chemical Engineering Journal [52]
9	Heavy metal removal from aqueous solutions using engineered magnetic biochars derived from waste marine macro-algal biomass	Son, EB	Cd^2+^, Cu^2+^, and Zn^2+^	248	2018	Science of the Total Environment [53]
10	Preparation of ultrafine magnetic biochar and activated carbon for pharmaceutical adsorption and subsequent degradation by ball milling	Shan, DN	carbamazepine and tetracycline	231	2016	Journal of Hazardous Materials [54]

**Table 5:** The advantages and effects of different modification methods in removing pollutants.

Modification Method	Raw Materials	Target Pollutants	Modification Reagent	Modification Conditions	Mechanisms	Advantage	Effect	Reference
Surface functional group modification	Palm fiber	Cd(II)	Iminodiacetic acid	T = 343 K t = 12 h	surface complexation	With the iminodiacetic acid modification, the oxygen-containing functional groups increased, resulting in the increase in adsorption capacity.	MBCI exhibited a high adsorption capacity of 197.96 mg/g at 323 K, 82.18% of its adsorption capacity after five consecutive cycles and magnetization value of 16.88 emu/g.	[89]
Acid and alkaline modification	Municipal solid waste (MSW)	As(V)	KOH	T = 500 °C t = 0.5 h	electrostatic interactions, surface complexes, metal precipitation	The alkaline treatment increases the surface area of biochar and changes in its porous structure, particularly increasing the presence of functional groups on the activated biochar surface.	The adsorption capacity of arsenic (As) by biochar was significantly improved following alkali treatment activation, resulting in a maximum adsorption capacity of 30.98 mg/g, which is over 1.3 times compared to the untreated biochar.	[90]
Biological modification	Rice straw	Cd(II), As(III)	Bacillus sp. K1	Mix 1 g of MBC with 40 mL of bacteria and shaken at 160 rpm and 25 °C for 12 h	Competition, synergy effects	The combination with Bacillus sp. K1 provided new biosorption sites such as amine and hydroxyl groups in the composite surface, which significantly increased the removal capability.	The removal rate of Cd(II) by composite materials is 230% higher than that of raw MBC. The binary system’s maximum adsorption capacities of Cd(II) and As(III) are 25.04 and 4.58 mg/g.	[91]
Metal element doping	Rice husk	Pb(II), Cd(II)	KMnO_4_	T = 600 °C t = 0.5 h	Redox interaction, surface complexation	KMnO_4_ modification successfully loaded manganese oxide on the surface of MBC, increasing oxygen containing functional groups. The adsorption performance of KMnO_4_-treated MBC for two heavy metals is almost not affected by Ionic strength and wet acid.	The maximum Langmuir adsorption capacity of KMnO_4_-treated MBC for Pb(II) reached 148 mg/g and for Cd(II) reached 79 mg/g, which is nearly 7 times that of raw MBC.	[92]
Non-metallic elements doping	Corn stalk	Cr(VI)	NH_3·_H_2_O	T = 300 °C t = 2 h	electrostatic attraction, complexion, precipitation, reduction and pore filling	Oxygen-containing and amino functional groups from N-doped biochar and polyethyleneimine (PEI) synergistically form a complex with Cr(III) ions by providing lone pair electrons, which form a coordinate covalent bond.	The maximum adsorption capacity for Cr(VI) is 183.02 mg/g. Furthermore, even after four adsorption–desorption cycles, the removal efficiency of Cr(VI) can still maintain 95.83%.	[75]

## Data Availability

Not applicable.
